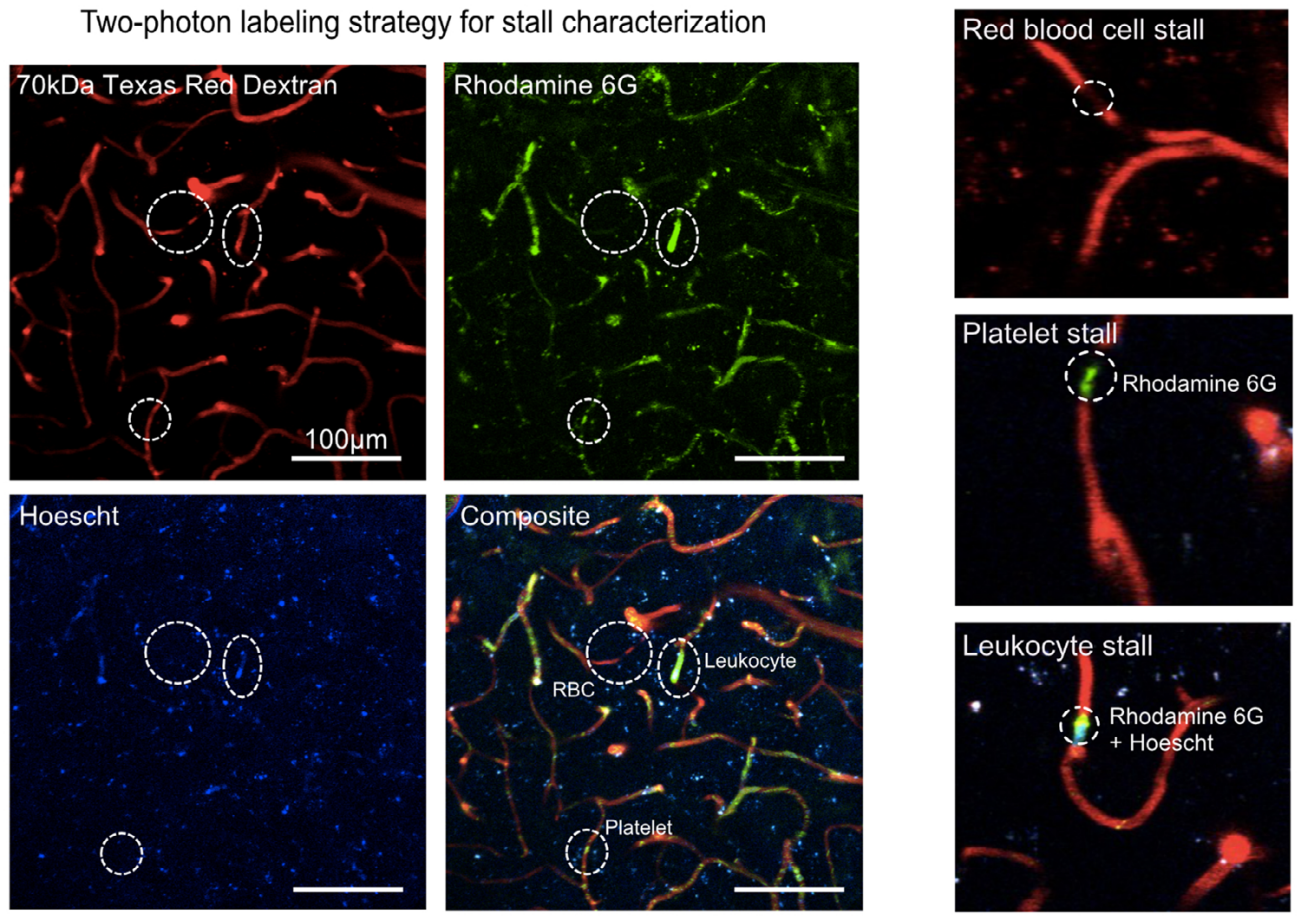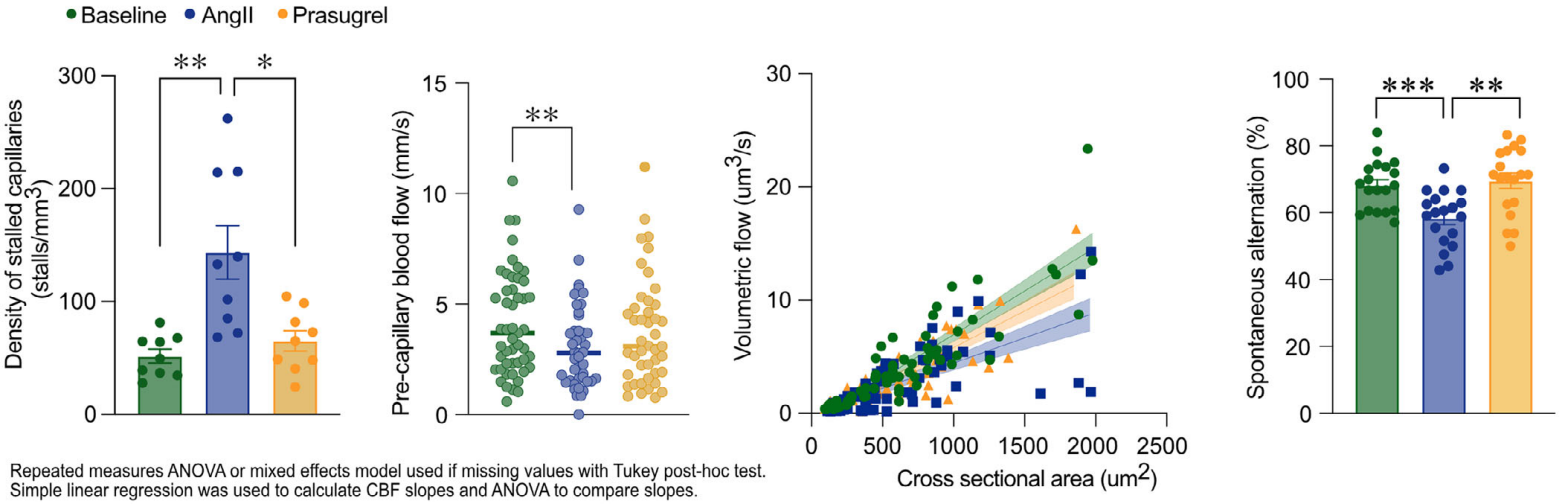# The anti‐platelet agent prasugrel reverses the cerebral blood flow deficit and memory impairment induced by hypertension in ApoE4‐positive mice

**DOI:** 10.1002/alz.088400

**Published:** 2025-01-03

**Authors:** Lianne J Trigiani, Nicole E Chernavsky, Rachel Kim, Robert F Hawkins, Nuri Hong, Keri Yamaguchi, Jonah Bernard, Amanda Huang, Gina H Bae, Daniel A Rivera, Nathaniel Allan‐Rahill, Michael Lamont, Costantino Iadecola, Nozomi Nishimura, Chris B Schaffer

**Affiliations:** ^1^ Cornell University, Ithaca, NY USA; ^2^ Weill Cornell Medical College, New York, NY USA

## Abstract

**Background:**

Several genetic and cardiovascular risk factors increase incidence of Alzheimer’s disease and related dementias (ADRD). Hypertension and the ε4 allele of apolipoprotein E (ApoE) are powerful drivers of cognitive impairment in ADRD. These risk factors are also associated with decreased cerebral blood flow (CBF). Experimental data suggest that the CBF reduction may be due to temporary capillary occlusions (capillary stalls) caused by circulating vascular and immune cells. To gain insight into how these risk factors may contribute to cognitive decline, we induced hypertension in mice expressing human ApoE3 or E4 (ApoE3‐ or E4‐TR mice), and focused on the resulting CBF and cognitive alterations, and their potential reversal by preventing capillary stalling.

**Methods:**

Male ApoE3 and 4‐TR mice (n = 9‐15/group) were assessed at three timepoints: Baseline, following two weeks of angiotensin II‐induced hypertension (AngII, 500ng/kg/min), and after an additional week of anti‐platelet treatment (prasugrel, 10mg/kg, daily). We tested spatial memory function in a Y‐maze, and imaged cortical microvascular flow using two‐photon microscopy.

**Results:**

At baseline, ApoE3 and 4‐TR mice showed similar memory performance, but with hypertension (∼25 mmHg increase in blood pressure) ApoE4‐TR mice exhibited a spatial working memory deficit, which coincided with a 26% decrease in capillary flow speed (4.11±0.3 vs. 3.05±0.29 mm/s, *p*<0.01), and a 1.8X increase in the incidence of non‐flowing capillaries. Most stalled capillaries contained only red blood cells in the stalled segment (57%), with some also containing leukocytes (30%), or platelets (12%) (Fig. 1). Treatment with prasugrel led to a 55% reduction in stalling (65±9 vs. 144±24 stalls/mm^3^), and a concomitant 23% increase in capillary blood flow (3.75±0.36 vs. 3.05±0.29 mm/s, *p* = 0.11), and significantly improved blood flow in penetrating arterioles that correlated with restored performance on the Y‐maze task (Fig. 2). Immunohistochemistry further revealed improved BBB integrity and reduced gliosis following prasugrel treatment.

**Conclusion:**

Paralleling human findings, we show that mice with the e4 allele of ApoE experience more deleterious consequences from hypertension, as compared to their e3 counterparts. Prasugrel treatment led to an almost complete recovery of these deficits, suggesting that anti‐platelet agents may be of therapeutic value by improving CBF and cognitive function in the presence of these risk factors.